# HbA1c Variability and the Risk of Dementia in Patients with Diabetes: A Meta-Analysis

**DOI:** 10.1155/2022/7706330

**Published:** 2022-01-31

**Authors:** Jingjing Song, Hongying Bai, Hui Xu, Yuanyuan Xing, Si Chen

**Affiliations:** Department of Neurology, The Second Affiliated Hospital of Zhengzhou University, Zhengzhou 450014, China

## Abstract

**Background:**

Variability of HbA1c has been related to the incidence micro and macrovascular complications in patients with diabetes. However, the association between of visit-to-visit variability of HbA1c and risk of dementia has not been fully determined. A meta-analysis was performed to comprehensively evaluate the above association.

**Methods:**

Medline, Embase, and Web of Science databases were searched for longitudinal follow-up studies comparing the incidence of dementia in diabetic patients with higher or lower variability of HbA1c. A random-effect model incorporating the potential heterogeneity among the included studies were used to pool the results.

**Results:**

Five retrospective studies with 577592 diabetic patients were included, and 99% of them were with type 2 diabetes mellitus (T2DM). With a mean follow-up duration of 6.3 years, 31963 patients had newly diagnosed dementia. Pooled results showed that diabetic patients with higher HbA1c variability was associated with higher risk of dementia, as evidenced by studies with coefficient of variation (CV: hazard ratio (HR): 1.06; 95% confidence interval (CI): 1.003–1.120; *p*=0.04; *I*^2^ = 47%) and standard deviation (SD : HR: 1.19; 95% CI: 1.06–1.32; *p*=0.002; *I*^2^ = 0%) of HbA1c in continuous variables, and CV of HbA1c (HR: 1.18; 95% CI: 1.08–1.28; *p* < 0.001; *I*^2^ = 31%) in categorized variables.

**Conclusions:**

Higher variability of HbA1c is associated with a higher incidence of dementia in patients with diabetes.

## 1. Introduction

Accumulating evidence suggests that diabetes may be a risk factor for cognitive impairment, and diabetes has been associated with higher risk of dementia [1–3]. Conventionally, persistent hyperglycemia evidenced by significantly increased plasma glycated hemoglobin (HbA1c) is well accepted as the most important cause of various complications in patients with diabetes [4, 5]. Subsequent studies demonstrated that besides persistent hyperglycemia, episodes of hypoglycemia associated with antidiabetic treatments are also associated with some adverse events and complications in patients with diabetes, including cognitive impairment [6, 7]. Interestingly, recent evidence suggests that higher glycemic variability, which reflects increased fluctuation in glycemia, may also be a strong risk factor for the incidence of complications in patients with diabetes [8–10]. Clinically, HbA1c is an indicator for the average glycemic status over 2-3 months, and variability of visit-to-visit HbA1c levels within months or years has been increasingly applied to reflect the level of long-term glycemic variability in diabetic patients [11, 12]. Previous studies have suggested that increased HbA1c variability, calculated as coefficient of variation (CV) or standard deviation (SD) of visit-to-visit HbA1c, may be independently associated with higher risk of vascular complications in patients with diabetes [13, 14]. However, it remains not fully determined whether HbA1c variability is also associated with a higher risk of dementia in diabetic patients [15–19]. Accordingly, in this study, we performed a meta-analysis to comprehensively evaluate the association between HbA1c variability and the risk of dementia in patients with diabetes.

## 2. Methods

The meta-analysis was performed in accordance with the MOOSE (Meta-Analysis of Observational Studies in Epidemiology) [20] and Cochrane's Handbook [21] guidelines. The protocol of this systematic review and meta-analysis was not prospectively registered.

### 2.1. Literature Search

Studies were identified via systematic search of electronic databases of PubMed, Embase, and Web of Science via the following terms: (1) “glycemic” OR “glyceamic” OR “glucose” OR “hemoglobin A1c” OR “A1C” OR “HbA1c”; (2) “variability” OR “variation” OR “fluctuation”; and (3) “dementia” OR “cognitive decline” OR “cognitive impairment” OR “cognitive dysfunction” OR “cognition” OR “Alzheimer” OR “Alzheimer's.” The search was limited to human studies published in English. The reference lists of related original and review articles were also analyzed using a manual approach. The final literature search was performed on August 21, 2021.

### 2.2. Study Selection

The inclusion criteria for the studies were as follows: (1) designed in longitudinal follow-up studies, including cohort studies, post hoc analysis of clinical studies, and nested case-control studies; (2) included patients with confirmed diagnosis of diabetes, including type 1 and type 2 diabetes mellitus (T1DM and T2DM); (3) visit-to-visit HbA1c variability was evaluated at baseline and quantified via the CV or SD of HbA1c; (4) evaluated the association between glycemic variability and incidence of dementia during follow-up; and (5) reported the hazard ratio (HR) for the above association with CV or SD of HbA1c analyzed as continuous variables (per 1-SD increment) or categorized variables (highest versus lowest category). Reviews, editorials, cross-sectional studies, studies with nondiabetic patients, studies evaluating acute glycemic variability, or studies irrelevant to the aim of current meta-analysis were excluded.

### 2.3. Data Extracting and Quality Evaluation

Literature search, data extraction, and quality assessment of the included studies were independently performed by two authors according to the predefined criteria. Discrepancies were resolved by consensus or discussion with the corresponding author. The extracted data included the name of first author, publication year, and country where the study was performed; study design characteristics; patient characteristics, including diagnosis of the patients, sample size, mean age, and sex; exposure characteristics, including parameters used for measuring of HbA1c variability at baseline (HbA1c-CV and/or HbA1c-SD), period for HbA1c variability calculation, and methods for HbA1c variability analysis; follow-up durations and outcomes reported; and confounding factors that were adjusted. The quality of each study was evaluated using the Newcastle-Ottawa Scale [22] which ranges from 1 to 9 stars and judges each study regarding three aspects: selection of the study groups, the comparability of the groups, and the ascertainment of the outcome of interest.

### 2.4. Statistical Analyses

We used HRs and their corresponding 95% confidence intervals (CIs) as the general measure for association between HbA1c variability at baseline and incidence of dementia during follow-up. Data of HRs and their corresponding stand errors (SEs) were calculated from 95% CI or *p* values and were logarithmically transformed to stabilize variance and normalized the distribution [21, 23]. Cochrane's *Q* test and estimation of *I* [2] statistic were used to evaluate the heterogeneity among the included cohort studies [24]. A significant heterogeneity was considered if *I* [2] > 50%. We used a random-effect model to synthesize the HR data because this model is considered as a more generalized method which incorporates the potential heterogeneity among the included studies [21]. Sensitivity analysis was used to evaluate the possible influence of each study on the pooled results. If at least ten datasets were included, the potential publication bias was assessed by funnel plots with Egger's regression asymmetry test [25]. A *p* value < 0.05 indicates a statistical significance. We used the RevMan (Version 5.1; Cochrane Collaboration, Oxford, UK) and Stata software for the meta-analysis and statistics.

## 3. Results

### 3.1. Literature Search

**Figure 1:**
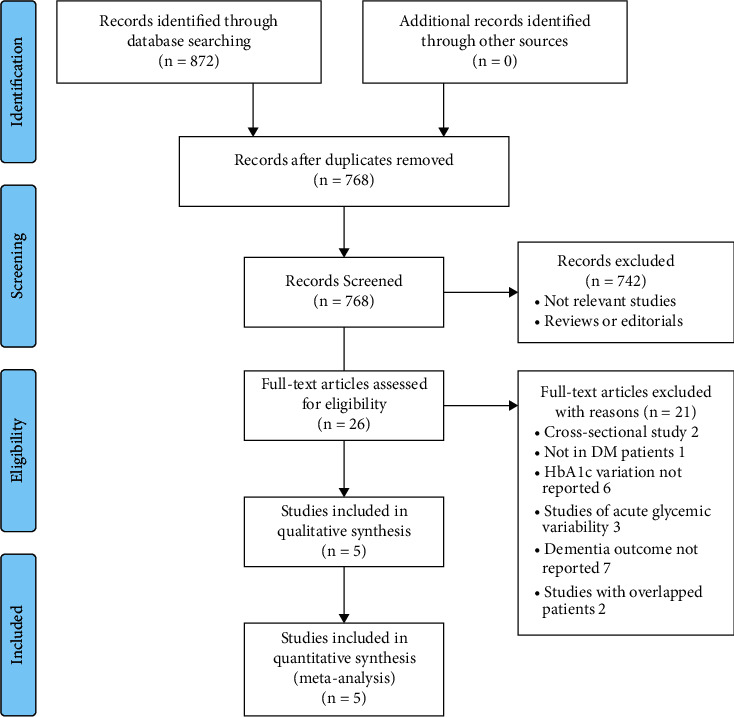
Flowchart of literature search.

The process of database search is shown in Figure 1. Briefly, 768 articles were found via initial literature search of PubMed, Embase, and Web of Science databases after excluding of the duplication, and 742 were further excluded through screening of the titles and abstracts mainly because they were not relevant to the purpose of the meta-analysis. Subsequently, 26 potential relevant records underwent full-text review. Of these, 21 were further excluded based on reasons shown in Figure 1. Finally, five studies were included [15–19].

### 3.2. Study Characteristics and Quality Evaluation

The characteristics of the included studies are given in Table 1. Overall, five studies, including four retrospective cohort studies [15, 16, 18, 19] and one post hoc analysis of the clinical study [17], were included. These studies were published between 2017 and 2021 and performed in China [15, 16, 18], Japan [17], and the United Kingdom [19]. All of the studies included patients with T2DM except for one study, which also included a small proportion (7%) of T1DM patients [18]. Overall, this meta-analysis included 577592 diabetic patients, and 99% of them were with T2DM. At baseline, glycemic variability was measured with HbA1c-CV and HbA1c-SD, which were analyzed as continuous variables in four studies [15, 17–19] and categorized variables in three studies [15, 16, 19]. One study reported the outcome of dementia related to Alzheimer's disease (AD) [15], and the other four studies reported the outcome of all-cause dementia [16–19]. With a mean follow-up duration of 6.3 years, 31963 patients had newly diagnosed dementia. Possible confounding factors, such as age, sex, smoking, alcohol drinking, obesity, baseline glycemic status, other comorbidities, and concurrent antidiabetic treatments, were adjusted to a varying degree among the included studies. The NOS scores of the included studies ranged from 6 to 8, indicating moderate to good study quality (Table 2).

### 3.3. HbA1c Variability and Risk of Dementia in Diabetic Patients

**Figure 2:**
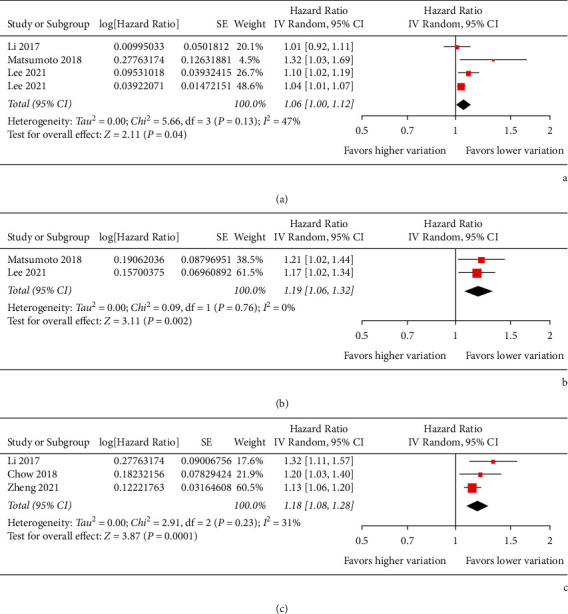
Forest plots for the meta-analysis of the association between HbA1c variability and risk of dementia in patients with diabetes. (a) Meta-analysis with HbA1c-CV analyzed as continuous variable. (b) Meta-analysis with HbA1c-SD analyzed as continuous variable. (c) Meta-analysis with HbA1c-CV analyzed as categorized variable.

Pooled results of four studies [15, 17–19] showed that higher HbA1c variability analyzed as HbA1c-CV in continuous variable was independently associated with higher risk of dementia in patients with diabetes (adjusted HR per SD: 1.06, 95% CI: 1.003–1.120, *p*=0.04; Figure 2(a)) with moderate heterogeneity (*p* for Cochrane's *Q* test = 0.13, *I*^2^ = 47%). Sensitivity by excluding the study reporting AD-related dementia [15] showed consistent result (adjusted HR per SD: 1.08, 95% CI: 1.001–1.168, *p*=0.04; *I*^2^ = 61%). Pooled results of two studies [17, 18] showed that higher HbA1c variability analyzed as HbA1c-SD in continuous variable was independently associated with higher risk of dementia (adjusted HR per SD: 1.19, 95% CI: 1.06–1.32, *p*=0.002; Figure 2(b)) with no significant heterogeneity (*p* for Cochrane's *Q* test = 0.76, *I*^2^ = 0%). Moreover, pooled results of three studies [15, 16, 19] showed that patients with the highest category of HbA1c-CV were with significantly higher risk of dementia as compared to those with the lowest category of HbA1c-CV (adjusted HR: 1.18, 95% CI: 1.08–1.28, *p* < 0.001; Figure 2(c)) with mild heterogeneity (*p* for Cochrane's *Q* test = 0.23, *I*^2^ = 31%). Sensitivity by excluding the study reporting AD-related dementia [15] also showed consistent result (adjusted HR: 1.14, 95% CI: 1.08–1.21, *p* < 0.001; *I*^2^ = 0%).

### 3.4. Publication Bias

**Figure 3:**
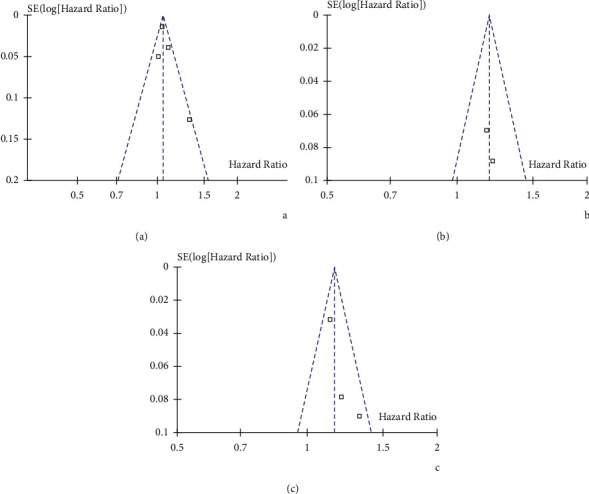
Funnel plots for the meta-analysis of the association between HbA1c variability and risk of dementia in patients with diabetes. (a) Funnel plots for studies with HbA1c-CV analyzed as continuous variable. (b) Funnel plots for studies with HbA1c-SD analyzed as continuous variable. (c) Funnel plots for studies with HbA1c-CV analyzed as categorized variable.

The funnel plots for the meta-analysis of the association between HbA1c variability and dementia risk as evaluated by HbA1c-CV and HbA1c-SD as continuous variable and HbA1c-CV as categorized variable are shown in Figures 3(a)–3(c). Tests for funnel plot asymmetry had little power for indicating publication bias since less than ten datasets were included. Egger's regression tests were not performed because of limited datasets for each outcome.

## 4. Discussion

In this meta-analysis, we pooled the results of five retrospective follow-up studies, and the results showed that higher visit-to-visit variability of HbA1c may be independently associated with higher risk of dementia in patients with diabetes. The robustness of the finding was validated by consistent results of meta-analyses with the variability of HbA1c analyzed as HbA1c-CV and HbA1c-SD as continuous variables and HbA1c-CV as categorized variables. These results suggested that besides persistent hyperglycemia and hypoglycemia episodes, increased long-term glycemic fluctuation may also be an independent risk factor for dementia in patients with diabetes.

As far as we know, this study is the first meta-analysis which summarized current understanding regarding the association between HbA1c variability and incidence of dementia in patients with diabetes. Before the interpretation of the results, some strengths of the meta-analysis should be highlighted. First, all of the included studies were longitudinal follow-up studies, which could therefore provide a temporal relationship between higher visit-to-visit variability of HbA1c and increased risk of dementia in patients with diabetes. Moreover, meta-analyses for the above association were separately performed according to the different parameters for HbA1c variability (HbA1c-CV and HbA1c-SD) and different analytic methods (continuous and categorized variables). Finally, for all of the included studies, the association between HbA1c variability and incidence of dementia was obtained in multivariate analyses after adjustment of possible confounding factors. Accordingly, a potential independent relationship between increased HbA1c variability and higher incidence of dementia in patients with diabetes could be retrieved. Taken together, these findings suggested that increased long-term glycemic fluctuation as evidenced by increased visit-to-visit variability of HbA1c may be an independent risk factor dementia in patients with diabetes. The results of the meta-analysis are consistent with findings of some previous studies which evaluated the association between glycemic variability and changes in cognitive function. An early study showed that higher acute glycemic peak indicated by the decreased level of 1,5-anhydroglucitol (1,5-AG) was independently associated with cognitive decline and dementia in community-based population of the Atherosclerosis Risk in Communities study [26]. In addition, a recent prospective population-based cohort study also suggested a significant association between HbA1c variability and cognitive decline among the nondiabetic population [27]. It has to be mentioned that these two cohort studies were performed in general population rather than in patients with diabetes, which may suggest that the association between glycemic fluctuation and cognitive impairment is not restricted to patients with diabetes. Some mechanisms could be proposed underlying the above association. For example, increased glycemic fluctuation has been associated with vascular complications of diabetes and atherosclerosis of cerebral arteries, and cerebral ischemia has been identified as major risk factors for cognitive impairment in these patients [28]. A recent study showed that glucose fluctuation is significantly associated with severe internal carotid artery siphon stenosis in T2DM patients [29], a known cause of vascular dementia. In addition, increased glycemic fluctuation is related to the severity of oxidative stress, a major pathogenesis and therapeutic target for AD-related dementia [30, 31]. Moreover, T2DM patients with higher HbA1c variability, despite of a possible suitable average HbA1c, may be more likely to suffer from recurrent hypoglycemic episodes, which have been recognized as a risk factor for cognitive impairment, particularly in the elderly [32]. Besides, glycemic fluctuation has been related to brain atrophy [33], altered microglial activity [34], and some molecular changes reflecting the degeneration of the brain regions related to cognitive deficits [35]. Our results, together with these findings, suggested the possible clinical significance of tightened and stable glycemic control in patients with diabetes, particularly for those at higher risk for dementia.

Our study has limitations. First, studies available for the meta-analysis were retrospective, which may be confounded by the recall or selection biases. Therefore, prospective cohort studies are needed for validation. Second, limited datasets were available for each metrics of HbA1c variability, and we were unable to evaluate the influences of study patient or study characteristics on the association, such as the age, sex, comorbidities, and follow-up durations. Large-scale prospective studies are also warranted for further investigation. Besides, we did not search for grey literatures or make contact with the related researchers for possibly unpublished data, which may also lead to additional bias of the systematic review. In addition, almost all studies included T2DM patients. Accordingly, the possible association between HbA1c variability and risk of dementia in T1DM patients should be evaluated in future studies. Moreover, although studies with multivariate analysis were included, we could not exclude the existence of residual factors that may affect the association, such as the concurrent use of antidiabetic medications that may reduce glycemic fluctuation. Furthermore, we could not determine the possible influence of publication bias on the results since only five studies were included because tests for funnel plot asymmetry and Egger's regression analysis could not be performed. Finally, a causative relationship between HbA1c variability and dementia could not be derived based on our study because it is a meta-analysis of observational studies. Clinical trials may be considered to evaluate whether reduction of glycemic fluctuation could reduce the incidence of dementia in diabetic patients.

In conclusion, results of the meta-analysis showed that increased HbA1c variability in patients with diabetes was significantly associated with higher risk of dementia. Glycemic fluctuation should be considered as a risk factor for dementia in diabetic patients, which should be considered in the determination of optimal hypoglycemic regimens in these patients.

## Figures and Tables

**Table 1:** Characteristics of the included studies.

Study	Country	Design	Participants	Sample size	Mean age (years)	Male (%)	GV measurement	GV parameter analysis	Period for GV calculation (years)	Follow-up duration (years)	Dementia outcome reported	Outcome validation	Patients with dementia	Variables adjusted
Li, 2017 [15]	China	R	T2DM patients aged 60 years or above	16706	69.2	44.6	HbA1c-CV	T3 : T1 or continuous	1 year	8.9	Dementia caused by AD	ICD-9 codes	831	Age, sex, FPG, HbA1c, smoking, alcohol consumption, DM duration, obesity, comorbidities, and concurrent medications
Chow, 2018 [16]	China	R	T2DM patients aged 60 years or above	75348	71.5	46./8	HbA1c-CV	T3 : T1	5 years	6	Overall dementia	Dementia with pharmacotherapy	1424	Age, sex, FPG, HbA1c, smoking, alcohol consumption, DM duration, BMI, comorbidities, and concurrent medications
Matsumoto, 2018 [17]	Japan	R	T2DM patients	2450	65	55	HbA1c-CV or HbA1c-SD	Continuous	NR	11.4	Overall dementia	Dementia with pharmacotherapy or hospitalization	129	Age, sex, FPG, HbA1c, smoking, alcohol consumption, DM duration, BMI, comorbidities, and concurrent medications
Lee, 2021 [18]	China	R	DM patients (T2DM: 93%)	25186	63	50	HbA1c-CV or HbA1c-SD	Continuous	NR	10	Overall dementia	ICD-9 codes	952	Age, sex, and comorbidities
Zheng, 2021 [19]	UK	R	T2DM patients aged 50 years or above	457902	64.5	52.1	HbA1c-CV	Q4 : Q1 or continuous	3 years	6	Overall dementia	Dementia with pharmacotherapy or hospitalization	28627	Age, sex, calendar year, and region, smoking status, BMI category, history of comorbidities, duration of DM, and concurrent medications

GV, glycemic variability; R, retrospective; DM, diabetes mellitus; T2DM, type 2 diabetes mellitus; T1DM, type 1 diabetes mellitus; FPG, fasting plasma glucose; HbA1c, hemoglobin A1c; SD, standard deviation; CV, coefficient of variation; T, tertile; *Q*, quartile; AD, Alzheimer's disease; ICD, international classification of diseases; BMI, body mass index.

**Table 2:** Details of study quality evaluation via the Newcastle-Ottawa Scale.

Study	Representativeness of the exposed cohort	Selection of the nonexposed cohort	Ascertainment of exposure	Outcome not present at baseline	Control for age	Control for other confounding factors	Assessment of outcome	Enough long follow-up duration	Adequacy of follow-up of cohorts	Total
Li, 2017 [15]	0	1	1	1	1	1	1	1	1	8
Chow, 2018 [16]	0	1	1	1	1	1	1	1	1	8
Matsumoto, 2018 [17]	0	1	0	1	1	1	1	1	1	7
Lee, 2021 [18]	0	1	0	1	1	0	1	1	1	6
Zheng, 2021 [19]	0	1	1	1	1	1	1	1	1	8

## Data Availability

The data used to support the findings of this study are available from the corresponding author upon request.

## References

[1] Biessels G. J., Despa F. (2018). Cognitive decline and dementia in diabetes mellitus: mechanisms and clinical implications. *Nature Reviews Endocrinology*.

[2] Srikanth V., Sinclair A. J., Hill-Briggs F., Moran C., Biessels G. J. (2020). Type 2 diabetes and cognitive dysfunction-towards effective management of both comorbidities. *The Lancet Diabetes & Endocrinology*.

[3] Biessels G. J., Whitmer R. A. (2020). Cognitive dysfunction in diabetes: how to implement emerging guidelines. *Diabetologia*.

[4] (2020). 6. Glycemic targets: standards of medical care in diabetes-2020. *Diabetes Care*.

[5] Shen Y., Shi L., Nauman E. (2020). Association between hemoglobin A1c and stroke risk in patients with type 2 diabetes. *Journal of Stroke*.

[6] Mattishent K., Loke Y. K. (2016). Bi-directional interaction between hypoglycaemia and cognitive impairment in elderly patients treated with glucose-lowering agents: a systematic review and meta-analysis. *Diabetes, Obesity and Metabolism*.

[7] Mattishent K., Loke Y. K. (2021). Meta-analysis: association between hypoglycemia and serious adverse events in older patients treated with glucose-lowering agents. *Frontiers in Endocrinology*.

[8] Smith-Palmer J., Brändle M., Trevisan R., Orsini Federici M., Liabat S., Valentine W. (2014). Assessment of the association between glycemic variability and diabetes-related complications in type 1 and type 2 diabetes. *Diabetes Research and Clinical Practice*.

[9] Nalysnyk L., Hernandez-Medina M., Krishnarajah G. (2010). Glycaemic variability and complications in patients with diabetes mellitus: evidence from a systematic review of the literature. *Diabetes, Obesity and Metabolism*.

[10] Gorst C., Kwok C. S., Aslam S. (2015). Long-term glycemic variability and risk of adverse outcomes: a systematic review and meta-analysis. *Diabetes Care*.

[11] Bergman M., Abdul-Ghani M., DeFronzo R. A. (2020). Review of methods for detecting glycemic disorders. *Diabetes Research and Clinical Practice*.

[12] Kovatchev B. (2019). Glycemic variability: risk factors, assessment, and control. *Journal of Diabetes Science and Technology*.

[13] Sun B., Luo Z., Zhou J. (2021). Comprehensive elaboration of glycemic variability in diabetic macrovascular and microvascular complications. *Cardiovascular Diabetology*.

[14] Picconi F., Di Flaviani A., Malandrucco I., Giordani I., Frontoni S. (2012). Impact of glycemic variability on cardiovascular outcomes beyond glycated hemoglobin. Evidence and clinical perspectives. *Nutrition, Metabolism and Cardiovascular Diseases*.

[15] Li T.-C., Yang C.-P., Tseng S.-T. (2017). Visit-to-Visit variations in fasting plasma glucose and HbA1cAssociated with an increased risk of Alzheimer disease: Taiwan diabetes study. *Diabetes Care*.

[16] Chow W. S., Lee P. C. H., Woo Y. C. (2018). Glycemic variability predicts the development of dementia in type 2 diabetes. *Journal of Diabetes Investigation*.

[17] Matsumoto C., Ogawa H., Saito Y. (2018). The association of visit-to-visit blood pressure and blood glucose variability and incidence of dementia in patients with type 2 diabetes mellitus: insights from the JPAD2 cohort study. *Circulation*.

[18] Lee S., Zhou J., Wong W. T. (2021). Glycemic and lipid variability for predicting complications and mortality in diabetes mellitus using machine learning. *BMC Endocrine Disorders*.

[19] Zheng B., Su B., Price G., Tzoulaki I., Ahmadi-Abhari S., Middleton L. (2021). Glycemic control, diabetic complications, and risk of dementia in patients with diabetes: results from a large U.K. Cohort study. *Diabetes Care*.

[20] Stroup D. F., Berlin J. A., Morton S. C. (2000). Meta-analysis of observational studies in EpidemiologyA proposal for reporting. *JAMA*.

[21] Higgins J., Green S. (2011). *Cochrane Handbook for Systematic Reviews of Interventions Version 5.1.0*.

[22] Wells G. A., Shea B., O’Connell D. (2010). The newcastle-ottawa scale (NOS) for assessing the quality of nonrandomised studies in meta-analyses. http://www.ohri.ca/programs/clinical_epidemiology/oxford.asp.

[23] Jiang P., Chen Y., Liu B. (2021). Prognostic efficacy of tumor-stroma ratio in women with breast cancer: a meta-analysis of cohort studies. *Frontiers in Oncology*.

[24] Higgins J. P. T., Thompson S. G. (2002). Quantifying heterogeneity in a meta-analysis. *Statistics in Medicine*.

[25] Egger M., Smith G. D., Schneider M., Minder C. (1997). Bias in meta-analysis detected by a simple, graphical test. *BMJ*.

[26] Rawlings A. M., Sharrett A. R., Mosley T. H., Ballew S. H., Deal J. A., Selvin E. (2017). Glucose peaks and the risk of dementia and 20-year cognitive decline. *Diabetes Care*.

[27] Yu Z.-B., Zhu Y., Li D. (2020). Association between visit-to-visit variability of HbA1c and cognitive decline: a pooled analysis of two prospective population-based cohorts. *Diabetologia*.

[28] Murthy S. B., Jawaid A., Qureshi S. U. (2010). Does diabetes mellitus alter the onset and clinical course of vascular dementia?. *Behavioural Neurology*.

[29] Eto F., Washida K., Matsubara M. (2021). Glucose fluctuation and severe internal carotid artery siphon stenosis in type 2 diabetes patients. *Nutrients*.

[30] Papachristoforou E., Lambadiari V., Maratou E., Makrilakis K. (2020). Association of glycemic indices (hyperglycemia, glucose variability, and hypoglycemia) with oxidative stress and diabetic complications. *Journal of Diabetes Research*.

[31] Butterfield D. A., Halliwell B. (2019). Oxidative stress, dysfunctional glucose metabolism and Alzheimer disease. *Nature Reviews Neuroscience*.

[32] Chen Y.-x., Liu Z.-r., Yu Y., Yao E.-s., Liu X.-h., Liu L. (2017). Effect of recurrent severe hypoglycemia on cognitive performance in adult patients with diabetes: a meta-analysis. *Current Medical Science*.

[33] Cui X., Abduljalil A., Manor B. D., Peng C.-K., Novak V. (2014). Multi-scale glycemic variability: a link to gray matter atrophy and cognitive decline in type 2 diabetes. *PLoS One*.

[34] Hsieh C.-F., Liu C.-K., Lee C.-T., Yu L.-E., Wang J.-Y. (2019). Acute glucose fluctuation impacts microglial activity, leading to inflammatory activation or self-degradation. *Scientific Reports*.

[35] Joshi M., Shah D. P., Krishnakumar A. (2021). Extreme glycemic fluctuations debilitate NRG1, ErbB receptors and Olig1 function: association with regeneration, cognition and mood alterations during diabetes. *Molecular Neurobiology*.

